# Extra Supplement—The Nursing Mirror

**Published:** 1890-08-09

**Authors:** 


Being the Extra Nursing Supplement of "The Hospital" Newspaper.

All Contributions for this Supplement should be addressed to the Editor, The Hospital, 140, Strand, London, W.C., and should have the word "Nursing" plainly written in left-hand top corner of the envelope.

### En Passant.

**The London Hospital.**—It has been moved and seconded, and unanimously carried: "That the House Committee desire to express their entire and unabated confidence in their matron, and their sympathy with her in the trouble and anxiety to which she has been subjected owing to the attacks recently made upon her."

**A Russian Story.**—Perhaps, in spite of potted lobster, we are better off in England than we understand. Take the following instance of a possible fate in Russia, gleaned from the *Fortnightly Review*; "The daughter of a Russian official wishes to study medicine and obtain a midwife's certificate. Her father discourages her in every way, but in vain. She leaves home without his permission, and goes to one of the university towns to study. Her father writes a letter to the police, asking that she be sent home at once, and she is sent as a convict—hungry, naked, insulted, deflowered. In time only her dead body reaches her native place—perhaps not even that."

**Massage.**—Too much stress cannot be laid on the culpable error of those who ought to know better in sending to an invalid requiring massing a nurse who has never had a lesson, but has seen it performed once or twice on a fellow nurse by one who, in her turn, had seen it done in hospital. What is the result? The nurse is thoroughly exhausted, because in her ignorance she has exerted every muscle in her body, and she leaves her unfortunate patient simply an "animated bruise," after having paid a liberal fee for massing. With perfect massing a bruise cannot be given, and as it is acknowledged on all sides that massage is a panacea for so much suffering, no pains should be spared to enhance instead of depreciate its value. A letter just received from Kimberley says: "You will be pleased to hear I have a great number of massage cases, and am so glad I learnt from Mrs. Creighton Hale before leaving. I like Kimberley Hospital immensely. There is plenty of work out here, good salaries, good food, and rooms to yourself away from the hospital." Other nurses abroad have told us how useful they have found a good knowledge of massage.

**Notes from America.**—St. Luke's Hospital Training School for Nurses in New York graduated its first class on June 28th. Six ladies received diplomas, and two of them will remain in the service of the hospital. The male nurses of the Mills School announce that they have no intention of working 18 hours a-day. Twelve hours is all they consent to work, and their example should be followed by private women nurses. The fifteenth meeting of the Bellevue Guild of Nurses was held on June 24th, and there were some pleasant songs and recitations. The members now number 79, and their object is to nurse the poor when otherwise disengaged, to form a nurses' club, and to encourage one another in work. The badge is a silver cross, with "Bellevue" on one side and "Ready, aye Ready!" on the other. Miss Snively, the lady superintendent of Toronto General Hospital, has been delivering a series of ambulance lectures to the public of that town. The Episcopal Hospital of Philadelphia lately awarded diplomas to eleven nurses; the proceedings were partly religious, Bishop Whittaker awarding the diplomas in the chapel in the middle of a service of song and prayer.

**How She Got Off.**—The *Globe* is responsible for the following story, which savours somewhat of Sairey Gamp days:—

*Judge:* Eliza Wilcox, you were drunk last night. What do you work at?

*Eliza:* I'm a nurse at the Riverside Hospital, taking care of small-pox patients.

*Judge* (*with alacrity*): Here, Eliza, you jes get.

And she "got."

**Portsmouth District Nurses.**—A special meeting of the subscribers to the Portsmouth Society for Nursing the Sick Poor was lately held. The society was founded seven years ago under the auspices of Admiral and Lady Hornby, but lately has fallen upon evil days, is unable to procure funds, and has been hampered by incompetent helps. The extraordinary way in which the meeting was carried out was sufficient proof that, however well meaning the subscribers were, they were not a methodical body. First it was resolved that the executive should in future consist of twelve ladies nominated by the meeting, which was passed by 22 votes to 19. Next it was resolved that the Association should not be wound up, but that the organisation of the executive committee be reconstituted. Ultimately the council were empowered to wind up the Association or reconstitute it as they deemed expedient.

**The Spirit of Discontent.**—Is it true that an evil, carping spirit is increasing amongst nurses? That so much has been done for them, that they are becoming like spoilt peevish children? Such are the complaints made in the public press, and in private letters sent to our office. The *Daily News* says:—"The woman whose soul, like that of Brother Jake Tobin's, is 'sot on chicken-fixins', is not likely to be a good and sympathetic nurse to the patients of the London hospitals, many of whom bear on their faces the traces of slow starvation." A letter from C. M. A. runs:—"You have patted those—I was going to say dratted—nurses on the back quite enough. I know them; perfect nuisances in a house, and utterly ruined by new high and mighty notions." It is a serious thing if indeed this spirit of grumbling is really wide spread; we had hoped that the late nauseous exhibition of it was merely accidental, and that most nurses would regard with disgust this prominence given to potted lobster and stale eggs. Where the food of a nurse is insufficient or bad, let her complain to the hospital committee; but if it is plentiful and plain let her rest contented, or else go back home to the flesh-pots of Egypt. You cannot be a good nurse if your thoughts are all for self; if you are everlastingly thinking your food is not good enough, your hours are too long, your wages are too small, your holidays are too short. Spare an occasional thought for your patients, please, nurses! Air your grievances at a proper time, but remember that there is also a time to keep silence. This perpetual whining for "more" is evidently becoming irritating to the public; it will soon forget that it ever thought nurses ministering angels, and unless the spirit of discontent is checked in our ranks, we will be regarded as an arrogant, assertive class of women, to be dreaded, not loved. It is very disheartening at this time to have to write thus; but when five old probationers try to injure, by their selfish grumbling, one of the grandest and greatest of metropolitan charities, which succours 111,000 of the sick poor yearly, it becomes our duty to speak plainly, and to ask all nurses to be very careful not to ally themselves with those who are bringing discredit on nurses as a body.

### Sketches of Insanity.

By Francis H. Walmsley, M.D. (Senior Assistant Medical Officer of Leavesden Asylum).

### VI.—MELANCHOLIA.

"Oh! who can speak the vigorous joys of health,
Unclogged the body, unobscured the mind?"—Thomson.

There exists a close connection between high vitality and pleasurable feeling, and between lowered vitality and painful feeling. The whole expression of a man in good health and spirits is exactly the opposite of one suffering from depression and sorrow; in the one case there is manifested bodily energy and mental activity; the individual is cheerful, gay, and animated, the mental operations are carried on with strength, vivacity, and rapidity. In depression and gloom all is reversed, the individual is sad, miserable, and dejected; his capability of mental effort is enfeebled, his muscular movements denote languor, prostration, and inactivity.

In melancholia the vital energies are sunk below the normal pitch, there is under-action of the whole organism and of its parts, evidenced by impairment or abolition of mental energy, by emotional disturbance of a painful character, and by relaxation of all the muscles of the body, small and large, hence a general stooping and collapse of the figure. The failing supply of nerve force is manifested in the mental pain, and general feeling of ill-being and in the slow and torpid movements.

An attack of melancholia generally comes on gradually; on comparing the individual with his former self it is remarked that he manifests some strangeness of conduct, he wears an anxious, worn, irresolute expression; he is more quiet and dull than usual, he shows loss of interest in his usual occupations; the enjoyments of life give him no pleasure, there is a disposition to seclusion, he displays suspicion and distrust, an alteration of affection towards near and dear relatives, and general dislike to others without adequate cause; there is a profound sense of painful depression, the patient has lost his self-confidence, he manifests indolence, and general indifference; revels in nothing, writes to nobody, shuns all exertion, a feeling of oppression, anxiety, dejection, and gloom has taken complete possession of him; he takes up morbid fancies about his health, often based on uneasy bodily sensations, and disorders of the digestive organs; frequently he is strenuous in his resistance to taking food; he becomes a prey to sorrow, trivial circumstances easily move him to tears, he disregards all consolation and repulses those who strive to solace him; he passes his hours in silence and in the most gloomy forebodings of temporal and eternal punishment; for him life has lost its freshness, and the relish for existence has vanished.

To those suffering from melancholia, intellectual efforts are oppressive, thought becomes dreary, monotonous, and painful, ideas present themselves sluggishly to the mind, they abandon themselves to a morbid self-watching; they conjure up delusions of persecution, of conspiracies and plots among relatives or friends] going on secretly against them. We know how refractory to all sane reasons are men with false ideas; logical reasoning avails not, thus they say they are ruined, robbed by everyone, poisoned, and even if one should reason with them, proof in hand, respecting the folly of their apprehensions, and reassure them in a thousand ways, they continually return to it, they are perpetually complaining, they incessantly repeat the same phrases, the same vague apprehensions of impending calamity; the delusions which they develop are of infinite variety, and generally correspond to the feeling of the sufferer; thus they believe they are the victims of unseen agencies working electricity, that they are followed by spies, or by the police, they entertain ideas of their own unworthiness, that they have disgraced their family, have committed some inexpiable crime or unpardonable sin, that they are forsaken of God, and doomed to eternal punishment. With very many the ruling propensity is that of self-destruction, an ungovernable morbid impulse impels the patient to commit suicide. There is either an entire absence of motive for the act, or the motive is based on a delusion; many imagine they hear voices from Heaven, which it is impious to resist, commanding them to put an end to their lives; in some cases their determination is so well concealed, that no suspicion exists until the design is carried out. Patients so affected require the closest watching, and those having charge of them should ever bear in mind "How oft the sight of means to do ill deeds makes ill deeds done."

In the more acute forms of the malady, the outbreak—melancholic frenzy—is the outcome of an anxious terror, the expression of the features, the acuteness of the senses, and the activity of the intellect, is an extremely virulent kind of misery or anguish, originating in pain, apprehension, and uncertainty; the patient is terror-stricken, is restless and agitated; he displays a painful eagerness to escape from some persecuting foe, his cries, attitudes, and motions express strongly his mental pain; he is in incessant movement, paces ceaselessly up and down the room, wringing his hands in an agony of despair; under the influence of this vague terror, he shouts, groans, or weeps loudly, his grief finding in tears and cries satisfaction and relief. He displays resistance and violence to those about him, the outward expression of his efforts to surmount the danger by which he believes he is encompassed.

*(To be continued.)*

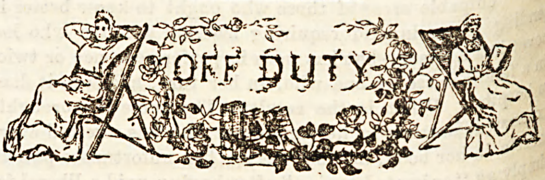

### Dr. Tarrant's Mistake.

*(Continued from page* lxxvii.*)*

Dr. Tarrant stared at No. 20 for at least ten minutes without speaking; then muttering something under his breath, removed the bandages and re-examined the wound carefully, making measurements of the head with his fingers. Finally he called for a chair, and sitting down quietly watched his patient. Long after all the dressers, &c., had gone, he sat watching. The nurse subsequently said that he fetched a case of instruments and undertook a careful examination of the man's head, but without attempting any operation. In fact Dr. Tarrant never went to bed that night, and only left the ward for a few minutes at a time. The next morning he appeared pale and jaded, but with the satisfied expression of a man who has solved some difficult problem. At eleven o'clock he made his appearance in the operating theatre dressed in the usual brown holland coat and operated on an unimportant case with his usual coolness and skill. There were not many men present, as the case, one of simple trepanning, was of no special interest. When, however, the patient had been removed from the table in the well of the room instead of retiring promptly according to his custom, Dr. Tarrant looked quickly round at those present and began the following astounding address:—

"Gentlemen, a case has recently come under my notice which is certainly unprecedented in my own experience, and, I have every reason to believe, in the record of surgery. The patient now occupying bed 20 in the accident ward, a young man of unknown name, was brought in yesterday afternoon suffering from a bullet wound nearly in the centre of the temporal bone. You are all of you aware, or at least you ought to be aware, that the front of the skull is entirely occupied by the fore part of the two hemispheres of the brain proper. The brain proper is, of course, the seat of the intellect. At the base and back of the skull between the two hemispheres and entirely covered by them lies the cerebellum, whose action I may compare to that of the balance-wheel of a watch, it regulates and co-ordinates movements. You will see that it is impossible for a bullet striking the forehead to reach the cerebellum without passing through one or both of the hemispheres, and I need hardly say that any bullet in passing through the brain would cause instant death. But what have we here? Here is a man whose sole injury is a bullet wound in the forehead, and yet he is suffering at the present moment from pressure on, or laceration of the cerebellum. He is quite conscious and intelligent, and therefore his brain has suffered no injury, but he has all the symptoms of aphasia and general ataxy. There is only one explanation—the patient must have an inverted brain, a brain turned completely upside down, so that the cerebullum is situated in the forehead and the hemispheres in the back and base of the skull. I need hardly point out to you the interest of such a complication; problems arise which an anatomist can barely conceive in the wildest freaks of his imagination. In what manner the spinal cord is inserted? What is the situation of the medulla oblongata? What modification do the optic, facial, and other nerves undergo? These are but a few of the questions of entrancing interest which a mere cursory consideration of the case suggests. Such a brain would be a perfectly unique preparation not to be paralleled in its abnormal development by the contents of any medical museum in the world. As regards the treatment of the case, it is much complicated by the uncertainty which necessarily arises. I must own myself completely in the dark as to the position of the different organs of the brain in this case, and, therefore, I have contented myself with taking merely superficial observations, and have not ventured even to probe for the bullet, because an irritation caused to the cerebellum at the present stage might cause convulsions, and a fatal termination of the case. I regret that you will be unable to see the patient, but he is in far too critical a condition."

Dismissing his audience with a slight bow, Dr. Tarrant left them to discuss in amazement the communication he had made to them. On the whole, such was his reputation for rigid, almost brutal accuracy, that they accepted his conclusions without much discussion. The more hardworking of them bringing out their anatomy manuals, proceeded to construct imaginary diagrams of the contents of No. 20's skull. The views of the more frivolous were fairly summed up in the remark of a youthful frequenter of music-halls.

"What an acrobat that man, with his brain upside down, would make! He'd feel like walking on his feet when he was standing on his head."

*(To be continued.)*

### A Note from Portugal.

Considering the ever increasing interests in sick nursing evinced by all classes, and which so often has the practical result of leading educated women to devote all their energies and intelligence to the especial training that will enable them to benefit their fellow mortals, whose bodily infirmities disable them, either temporally or permanently, from fighting the battle of life; it struck me that a short account of what a Portuguese lady is doing in the City of Oporto to alleviate one particular form of suffering among the poor around her, might not be without some interest to the readers of The Hospital.

I cannot claim any professional knowledge myself, but like everyone else have friends who have made nursing their life work, and on the other hand, know some whose circumstances or health quite prohibit this entire devotion, and who, therefore, do not attempt to do anything at all. These last perhaps, might be especially interested in what I have to tell. In Portugal, among the poor, there is nothing so common as for a child to be ruptured; the Portuguese lady to whom I have already referred has taken up this subject, after having gone through a medical course that has qualified her to treat children so afflicted with very great skill and success. She lives at her own home, and has one or two days a-week, when she sees her small patients at stated hours. I was told about her while on a recent visit to Oporto. A couple of years ago the baby of a relative of mine became ruptured after a very severe attack of dysentery while teething; the doctor attending him strongly advised his being taken to a seniora, who, he said, was particularly clever with cases of this description, and gave her address, saying it would most likely be only necessary to appoint a meeting and give some small present when the course of treatment was over. This lady never charged fees, as she wished more particularly to cure the children of the poor as a work of charity, the only difference made between the case of a poor person's child or one with parents in a position to pay being that with the latter the bandages were not supplied free. Acting on the doctor's advice, an appointment was duly made and the child was taken once a week to be bandaged by the seniora. Her arrangements were most simple; a small, rather barely furnished room, with a table set out with the necessary appliances, herself always ready at the appointed hour to attend to the child whom she managed most kindly and skilfully, arranging the bandages so quickly that as a rule the baby never cried at all while under her hands. It was entirely owing to this lady's treatment that the child was cured, the cure being, I am glad to say, a most complete one. It was only occasionally that the lady undertook these cases, wishing more especially to help people who were too poor to procure medical treatment for their children.

Thus living at her own home, and with absolutely no ostentation or assumption of superior skill, she was able to benefit her poorer neighbours in a manner far outweighing any amount of indiscriminate charity, that is, perhaps, even more general in Portugal than in England.

### Everybody's Opinion.

*[Correspondence on all subjects is invited, but we cannot in any way be responsible for the opinions expressed by our correspondents. No communications can be entertained if the name and address of the correspondent is not given, or unless one side of the paper only be written on.]*

### WORKHOUSE NURSES.

A Wandsworth Union Nurse writes: I am so sorry to see such a discouraging picture drawn of Union Infirmary life as there is in this week's Hospital. Our Board and also our senior officers, from the doctors (of whom we have three residents) downwards, try to make us as happy as ever they can. *Our* patients, with very few exceptions indeed—and we make up beds for nearly 1,000 I believe—are happy and contented, and think a great deal of their nurses. Our duties begin at 7 a.m., with an hour for dinner, and we usually have a good game at tennis (the doctors, steward, and matron joining us); half an hour for tea, going off duty and the night staff coming on at 7 p.m. We are allowed out until 10 every evening if we care to go; if not, we have a recreation room with piano and harmonium in it, where we often spend a very pleasant evening. Our food is good, though it might be varied a little more perhaps, and our salaries are not £20 as stated, but commence at £20, rising to

£26 by an increase of £2 each year, with indoor and outdoor uniform. People picture us as much to be pitied, but if they saw us at our work amongst our patients they would quickly change their tales. I am charge nurse of a female surgical floor, and my wards are bright with flowers, pictures, and plants. I think you will gather from this that our lives are not so gloomy as they are pictured.—[We did not mean to be discouraging. We have the greatest respect for those nurses who join the Workhouse Infirmary Nursing Association; and we know well that the Association cares for them to the best of its power. We hope more nurses will join the Association.—Ed.]

### AN AMERICAN NOTE.

Miss Dora Jones writes from Cincinnati:—"My sister and self, both American-trained nurses, but English by birth, have read The Hospital for the last year and enjoy it very much. Judging from what we have read, the position of a nurse here and in England is slightly different. Nursing Institutes are unknown (to us they seem very unjust); every private nurse is 'on her own hook.' There is no 'bureau' or nurses' register, consequently the poor unfortunates who need a nurse, after traversing enormous distances, with only two or three names (of course, all out), declare piteously that there are no nurses to be had. We are busy most of the time, at the salary of 20 dollars per week, but, as the Albany nurse wrote, it does not go very far, rent, board, etc., being very high. Far from being tired of 'talking shop,' we see no one to talk shop to, hardly ever meet with a fellow-worker, Doctors being very 'snubby,' much afraid of nurses encroaching on their prerogative—some of them anything but gentlemen. One wonders at times why they did not take up grammar with their medical studies. On the whole, I think the American nurse leads a freer but more isolated life than her English sister. When unemployed we relieve the monotony of existence by going to theatres, amusements, etc. But we have no clubs, no social réunion of nurses; no benevolent person 'takes us up.' We pursue the 'even tenor of our way,' a very small and unnoticed portion of the community."

### FOR OR AGAINST?

"A Member of St. Barnabas Guild" writes: I think it only fair to express my opinion as a nurse of the London Hospital with regard to the late charge brought against it. I went to a hospital supposed to be a model hospital in 1882, and I must candidly confess that I was almost starved there, and I paid £1 1s. per week besides paying for my washing and uniform. I only stayed there six months, and at the end of the year I went to the London, and I must say met with every kindness and consideration, and I think the nurses who grumbled at the living there ought to be starved. May I give you the day's food for an instance? Half-past six a.m., breakfast, boiled ham and coffee or tea; nine a.m., lunch, bread and butter and tea; one p.m., dinner, roast pork, onion sauce, greens, and potatoes, with jam pudding; four p.m., tea, bread and butter and tea; half-past nine p.m., supper, cheese, soup, or pudding and cold meat. There was also ale and porter, or milk, for any who liked it, and I don't think anybody ought to be discontented at this! Miss Lückes dismisses nurses without knowing why she does it. If a sister takes a personal dislike to a nurse, and likes to report her to the matron, her days at the hospital are numbered. Miss Lückes is indeed "Queen of the Nurses," but don't let us make out she's worse than she is. I, for one, feel very proud of the training I received there, and do not in the least regret being "overworked and underfed."

### Notes and Queries.

To Correspondents.—1. Questions may be written on post-cards. 2. Advertisements in disguise are inadmissible. 3. In answering a query please quote the number. 4. If a private answer is desired, a stamped addressed envelope must be forwarded.

Notice.—We must really ask our correspondents to be more particular in enclosing name and address. Constantly we receive queries or letters with no name attached.

### Answers.

A. B. (Gipsy Hill) must send address if she desires an answer.

*Constant Reader.*—The Secretary, St. John's Ambulance Association, St. John's Gate, Clerkenwell, E.C. You will find their classes of no use in becoming a professional nurse. You must train in a hospital or infirmary. Have you applied to the Workhouse Infirmary Nursing Association, 6, Adam Street, Strand? They would probably secure you the training you desire.

*F. J. D.*—We have already published one letter from Berry Wood. Your advantages are exceptional—what is true of one asylum is, alas! not true of all.

*L. W.*—We will give you an address next week; at present we have made no arrangements to receive parcels. We are very pleased to think you are working for us.

*M. D.*—Apply to Mrs. Creighton Hale, 46, Maddox Street, W.

*Nurse G.*—You will find the particulars your require in The Hospital for October 20th, 1888. Every month we publish a letter from Australia giving particulars of the various hospitals there. You must really look up the back numbers.

### Appointments.

[It is requested that successful candidates will send a copy of their applications and testimonials, with date of election, to The Editor, The Lodge, Porchester Square, W.]

Brookwood Asylum.—Miss Green has been appointed assistant matron at this asylum. Miss Green was trained at St. Thomas's Hospital. We wish her every success; it is a pleasure to know a trained nurse has been appointed to this post.

Government Hospital, Hong Kong.—Miss Mary A. Thompson, Miss Frances E. Higgin, and Miss Keturah E. Browne have been appointed sisters of wards at the above hospital. Miss Thompson trained at St. Marylebone Infirmary, Notting Hill, and is now sister there; Miss K. E. Browne also trained at St. Marylebone Infirmary; Miss Frances E. Higgin trained at Guy's Hospital, and is now sister at St. Marylebone Infirmary. These three ladies will proceed to their destination early in September, with Miss Eastmond, of the London Hospital, whose appointment as head sister of the Government Hospital, has been already announced.

### Vacancies.

The following vacancies are announced:

### Nurses.

Albert Hospital, Devonport; £25.

Bromley and Surbiton Nursing Institutions.

Birmingham and Midland Hospital for Women; £23.

Bishop's Stortford Nursing Institution; £25.

Borough Fever Hospital, Sheffield; £25.

Bournemouth Nursing Institute; £25.

Blackheath and Richmond Nursing Institutions.

Brixton Nursing Institution.

Cheltenham Nursing Institution.

Cottage Hospital, Harrow; £30.

Croydon Union; £22.

Church Deaconess' House, Chester; £24.

General Nursing Institute, W.C.

Hospital for Children, Pendlebury; £20.

Institution of Trained Nurses, Leicester; £25.

International Institution, Paris; £28.

Kent Nursing Institution; £30.

Levick Institution, Paris.

Liverpool Ear and Eye Hospital; £20.

Leith Hospital; £20.

Leith Hospital (night); £30.

Nursing Institution, Wimpole St.

Nurses' Home, Newcastle-on-Tyne.

Nurses' Home, Hull.

Nurses' Home, Bengeo, Herts; £30.

Nurses' Home, Truro; £25.

Nurses' Institute, Weston-super-Mare.

Oswestry and Ellesmere Cottage Hospital; £25.

Pembrokeshire Infirmary, S. Wales.

Shoreditch Infirmary; £20.

Shoreditch Infirmary (assist.); £17.

St. Helena Home, N.W.

St. Saviour's Infirmary, S.E.; £20.

St. John's Nurses' Institute, Southport; £25.

Tyrrel Hospital, Ilfracombe; £18.

Torquay Nursing Institution.

Victoria Home for Nurses, Bournemouth.

Warrington Infirmary (assist.); £18.

Workhouse Infirmary Nursing Association.

West Ham Hospital; £24.

### Probationers.

Bristol Hospital for Children.

Durham County Hospital.

Hospital for Chronic and Convalescent Children, N.

Hull Royal Infirmary.

Hospital for Diseases of Chest, City Road.

Kilmarnock Infirmary.

Kidderminster Infirmary.

Nurses' Institute, Canterbury.

Queen Victoria's Jubilee Institute (Scottish Branch).

St. Pancras Infirmary; £10.

St. Mary's Day Nursery, Plaistow.

West Ham Infirmary.

### District Nurse.

Queen's Nursing Institute, Cardiff; £30.

### Amusements and Relaxation.

### SECOND QUARTERLY WORD COMPETITION.

Commenced July 5th, 1890, ends Sept. 27th, 1890.

Three prizes of 15s., 10s., 5s., will be given for the largest number of words derived from the words set for dissection.

Proper names, abbreviations, foreign words, words of less than four letters, and repetitions are barred; plurals, and past and present participles of verbs, are allowed. Nuttall's *Standard* dictionary only to be used.

N.B.—Word dissections must be sent in WEEKLY not later than the first post on Thursday to the *Prize Editor*, 140, Strand, W.C., arranged alphabetically, with correct total affixed.

The word for dissection for this, the SIXTH week of the quarter, being

"FILBERTS."

| Names. | July 31st. | Totals. |
|---|---|---|
| Dulcamara | 85 | 165 |
| Tinie | 85 | 170 |
| Henri | 77 | 160 |
| Agamemnon | 85 | 168 |
| Patience | 85 | 167 |
| Brisbane | 59 | 87 |
| Nurse Hilda | 77 | 154 |
| Lightowlers | 85 | 161 |
| Jenny Wren | 81 | 152 |
| Rhoda | 75 | 137 |
| Annabel | — | 17 |
| Spare Moments | — | 19 |
| Qu'appelle | 85 | 163 |
| Nurse | — | 29 |
| A. B. C. | 37 | 69 |

